# Development of natural template‐based novel NIRF theranostic agents for Alzheimer’s disease

**DOI:** 10.1002/alz.095006

**Published:** 2025-01-09

**Authors:** gyan Prakash modi, Saroj kumar gupta, SRIKRISHNA kumar SARAPALLIE, Sarika kumar Gupta, HIMANSHU RAI

**Affiliations:** ^1^ Department of pharmaceutical engineering & technology, Varanasi, UP India; ^2^ All india institue of medical sciences, delhi, delhi India; ^3^ BHU, Varanasi, up India; ^4^ NII, delhi, delhi India

## Abstract

**Background:**

Alzheimer’s disease (AD) is a brain related neurological disorder characterized by gradual loss in memory along with other peripheral and central symptoms. The currently available treatments for AD provide only symptomatic relief without addressing the pathological hallmarks of the disease, therefore, neurodegeneration continues with these therapies.

**Method:**

The novel unique NIRF probes were designed, characterized with help of NMR techniques followed by biological evaluation against amyloid‐β (Aβ) and cholinesterases (ChEs) through various in‐vitro studies. The lead probe was tested in APP/PS1 mice, AD autopsy samples and AD drosophila models.

**Result:**

Given the potential role of amyloid‐β (Aβ) and cholinesterases (ChEs), we discovered unique dual functionality theranostic against these targets. The lead probe I‐43 showed a substantial stoke shift (225 nm), high affinity (K_d_ = 83 nM), deep NIR imaging contrast for Aβ aggregates, and sensing efficacy for AChE/BChE in in‐vitro AD models. The lead probe molecules have shown promising and selective Aβ aggregation detection ability in different AD models including transgenic AD mice model and AD patient autopsy samples.

**Conclusion:**

The unique ocular imaging pattern in the AD Drosophila model, strongly suggest that probes hold promise as a dependable indicator for rapid, noninvasive assessment of new therapeutic modulators or inhibitors in AD along with the improved cognitive function in a scopolamine‐induced amnesia model upon I‐43 treatment.

